# Population norms for the EQ-5D-5L for Hungary: comparison of online surveys and computer assisted personal interviews

**DOI:** 10.1007/s10198-024-01755-2

**Published:** 2025-02-21

**Authors:** Márta Péntek, Viktor Jáger, Áron Kincses, Áron Hölgyesi, Zsombor Zrubka, Petra Baji, Levente Kovács, László Gulácsi

**Affiliations:** 1 University Research and Innovation Center, Health Economics Research Center, Óbuda University, Budapest, Hungary; 2 Doctoral School of Innovation Management, Óbuda University, Budapest, Hungary; 3 Hungarian Central Statistical Office, Budapest, Hungary; 4 Institute of World and Regional Economics, University of Miskolc, Miskolc, Hungary; 5 Doctoral School of Molecular Medicine, Semmelweis University, Budapest, Hungary; 6 Musculoskeletal Research Unit, University of Bristol, Bristol, UK; 7 University Research and Innovation Center, Physiological Controls Research Center, Óbuda University, Budapest, Hungary

**Keywords:** Health-related quality of life, EQ-5D-5L, Population norms, Cross-sectional studies, Health surveys, I10

## Abstract

**Background and objectives:**

The aims of this study were to provide population norms for EQ-5D-5L in Hungary and investigate the differences in EQ-5D-5L normative data by survey mode, i.e. online surveys and computer assisted personal interviews (CAPI).

**Methods:**

A pooled database was built comprising six online (*N* = 7,034) and two CAPI (*N* = 3,020) population-based studies with the EQ-5D-5L. Descriptive statistics were performed. Multinominal logistic and linear regression analyses were applied to compare the online and CAPI samples. Traditional and machine learning regression tools were used to investigate the determinants of EQ-5D-5L index values.

**Results:**

‘No problems’ in any of the five EQ-5D-5L domains were reported by 33.9% (online) and 58.9% (CAPI) of the participants. Most problems were reported on the pain/discomfort domain in both study types (51.9% and 33.6%, respectively). Men and more educated respondents had significantly higher average EQ-5D-5L index values. EQ-5D-5L index values and EQ VAS scores were significantly higher in the CAPI sample, except in age groups 65–74 (no difference) and 75+ (online scores were significantly higher). Only 7–10% of variance in the EQ-5D-5L index values was explained by the variables survey mode, education, sex and age, with age having the largest and sex the smallest effect.

**Conclusions:**

EQ-5D-5L population norms derived from online and CAPI studies may differ significantly from each other. It is recommended to consider the survey mode, sampling and sociodemographic characteristics of the participants when choosing population norms as reference set. Further comparative studies investigating EQ-5D-5L population norms by different study designs and administration modes are encouraged.

**Supplementary Information:**

The online version contains supplementary material available at 10.1007/s10198-024-01755-2.

The EQ-5D instrument is among the most frequently used patient-reported measures to assess health-related quality of life impact of diseases and health interventions [1]. As a generic measure, EQ-5D can be used to assess and compare the health status of the population and different patient groups [2]. EQ-5D is a preference-based measure, societal utility values (preference weights) can be attached to all the health states described by its classification (descriptive) system. These utility values are used to calculate quality-adjusted life years (QALYs), in cost-utility analyses in health technology assessments (denominator when calculating the incremental cost-effectiveness ratios) [3]. EQ-5D can be also used to assess disease burden (health loss related to a disease) by comparing patients’ data with their counterparts from the general population. Population norms serve as reference data for this purpose, obtained from samples representative for the general population of a given country or jurisdiction.

The first EQ-5D version, the EQ-5D-3L, was developed in 1990 and a wealth of knowledge and population norms have been accumulated with this measurement tool over the past decades [4, 5]. In 2011, the EQ-5D-5L version was launched with the aim of improving the measurement properties of the original instrument [6]. Since then, intensive research activity has been observed around the EQ-5D-5L. More than 150 language versions came out in different modes of administration, including self-complete, proxy and telephone modes for paper and digital (e.g., Lime survey, Qualtrics, REDCap) platforms. Valuation studies, producing country-specific value sets, have been standardized allowing better comparability across studies and countries [7, 8]. The samples of the valuation studies (in which quotas are set by main demographic characteristics) are commonly used to provide population norms (e.g., Germany [9], Romania [10]) but other epidemiological studies have also been reported. However, little is known about how the study design, the administration mode and the platform used might impact the EQ-5D-5L population norms results. Stavem and colleagues compared a postal and a web survey with the original EQ-5D-3L version and found rather similar results both in terms of distribution of scores on the descriptive system and utility scores [11]. According to our best knowledge, comparative population norms analysis with the EQ-5D-5L was performed only in the USA by Jiang and colleagues in which a face-to-face and an online sample were matched [12]. In Hungary, population norms for the EQ-5D-5L have been recently published from one online study [13].

In the past years, our research group led several population-based online and computer-assisted personal interview (CAPI) studies in which quotas were set to guarantee representativeness for the adult Hungarian population by main sociodemographic characteristics, and the EQ-5D-5L was used to assess the health status of the respondents. It seemed worthwhile to reuse this rich dataset and investigate the data further. The primary aim of our study was, therefore, to develop EQ-5D-5L population norms for Hungary using large population samples. Secondarily, we aimed to analyse potential differences in EQ-5D-5L normative data obtained from online surveys and CAPI studies.

## Materials and methods

### EQ-5D-5L measurement tool

The EQ-5D-5L questionnaire comprises two parts, a descriptive system, and a visual analogue scale EQ VAS [6]. The descriptive system covers five general health domains, i.e., mobility, self-care, usual activities, pain/discomfort, anxiety/depression. In each domain, respondents are asked to indicate on a 5-level response scale (1-no, 2-slight, 3-moderate, 4-severe, 5-extreme problems) the one that best describes their health on that day. Based on the responses, altogether 3,125 different health states can be described (e.g., health state ʻ11111’ refers to no problems in any of the five domains, ʻ21111’ refers to slight problems only in mobility, ʻ55555’ means extreme problems in all the five domains). These 5-digit numbers can be converted into one single index using a utility value set established in a population-based study. This is the so-called EQ-5D-5L index value, that expresses the utility (preference weight, desirability) the society attaches to each health profile. In our study, we used the Hungarian EQ-5D-5L value set to calculate the index values, with a range of -0.848 and 1 (as 1 indicates full health, 0 is the state of death, negative values refer to health states that are considered worse than death) [14]. The EQ VAS is a 20 cm vertical scale with endpoints of 0 and 100 (worst and best imaginable health, respectively). Respondents are asked to indicate their current health (on the day of the completion of the questionnaire) on this numeric scale.

### Data sources

Eight cross-sectional studies conducted between 2018 and 2021 were involved in the analyses, out of which six were online surveys and two were CAPI. All the eight studies were completely independent from each other. Here we provide a summary of their methods that have been detailed in their original publications.

### CarerQol valuation study in Hungary – online survey

In this study, population tariffs for the CarerQol instrument, a preference-based measurement tool to assess care-related burden of informal caregivers, were obtained in a three-country study [15]. (Hereinafter: CarerQol study.) A cross-sectional online survey was performed between November 2018 and January 2019. Recruitment and data collection were performed by a professional survey company among a large online panel. The target sample size was 1,000 in Hungary. Quotas were set to obtain a representative sample for the adult Hungarian population by age, sex, educational level, type of settlement and region of residence up to 65 years of age, and authors aimed to achieve a fair representation of the Hungarian population aged 65 and over.

### Subjective health expectations of the general population in Hungary – online survey

This was a cross-sectional online survey conducted in Hungary in 2019 aiming to explore subjective expectations of the general population regarding health and happiness at future ages [16, 17]. (Hereinafter: Subjective expectations study.) Recruitment method, target sample size (*N* = 1,000) and quotas applied were the same as detailed above in the CarerQol study.

### Exploring eHealth literacy, shared decision making and patient reported experiences with outpatient care in Hungary – online survey

The study aimed to assess eHealth literacy of the Hungarian population, to detect the level of shared decision making in medical decisions and to explore patients’ experiences with healthcare [18–24]. (Hereinafter: eHealth literacy study.) A cross-sectional online survey was performed in Hungary, year 2019. Recruitment method, target sample size (*N* = 1,000) and quotas applied were the same as detailed above in the CarerQol and Subjective health expectations studies.

### Validation of the musculoskeletal health questionnaire (MSK-HQ) for Hungary – online survey

The MSK-HQ is a patient reported outcome measure developed to assess how MSK problems affect the life of patients living with diverse MSK conditions. The study’s aim was to develop and validate the Hungarian version of the MSK-HQ [25]. (Hereinafter: MSK-HQ study.) An online survey with cross-sectional design was performed in May–June 2020 in Hungary. Targeted sample size was 2,000 persons, the final sample size was 2004. Recruitment of respondents and data collection was performed by a survey company among members of an online access panel. Quotas were set for age, sex, educational level and type of settlement.

### Validation of the patient activation measure (PAM-13) for Hungary – online survey

PAM-13 is a self-reported outcome measure to assess patients’ knowledge, skill and confidence for self-management. This study aimed to produce and validate the PAM measurement tool for Hungary [26]. (Hereinafter: PAM study.) An online survey was conducted (April, 2020) recruiting respondents aged 40 years and older from a large online panel via quota-based sampling to ensure representativeness in terms of age groups, gender, education, geographic region and type of settlement. Target sample size was 900, finally 779 respondents with complete data were included in the analyses of PAM-13 validation study [26] but all the 900 respondents were included in our analyses as all relevant sociodemographic and EQ-5D-5L data were available. Both recruitment and data collection were performed by an online research company.

### Epidemiology of implantable medical devices in Hungary – online survey

In this study authors aimed to assess the epidemiology and patients’ knowledge of implantable medical devices (IMD) in Hungary [27]. (Hereinafter: IMD study.) A cross-sectional web-based survey study was carried out in July 2021, involving a sample (*N* = 1,400) of the Hungarian general population aged 40 years. Quota sampling was applied with quotas for sex, age, education and residency. Both the recruitment and data collection were performed by a survey company, reaching out an online access panel by emails.

### Hungarian population health survey – CAPI

This study (year 2019) aimed to measure the health status and wellbeing of the Hungarian adult population [28–30]. (Hereinafter: Population health study.) A survey company was contracted to perform the recruitment (random walk door-to-door recruitment method) and data collection. The target sample size was 2,000 persons, final sample size was 2,023 but three respondents were excluded from our analyses due to missing data. Quota sampling method was applied based on national population statistics to ensure representativeness in terms of sex, age group, settlement type and geographic region. Data collection was performed via CAPI. Paper-based self-completed versions of the EQ-5D-5L questionnaire was completed by the participants and these data were recorded subsequently in the electronic database.

### Valuation of the ICECAP-A questionnaire in Hungary – CAPI

The aim of this study was to develop population tariffs for the preference-based ICECAP-A capability wellbeing measurement tool for Hungary [31]. (Hereinafter: ICECAP-A valuation study.) A cross-sectional survey was conducted between May and June 2019 by a survey company, with target sample size of 1000, representative for the Hungarian adult population in terms of age, sex, and education. The survey methodology was CAPI, respondents completed the self-completed digital version of the EQ-5D-5L questionnaire.

### Creating a joint database

Person-level data from the eight studies were used to develop a joint database. Main sociodemographic variables (age, sex, type of residency, educational level), EQ-5D-5L descriptive system data and EQ VAS were extracted from each study. EQ-5D-5L index values were calculated based on the EQ-5D-5L health profiles (descriptive system) obtained from the original studies using the EQ-5D-5L value set for Hungary [14].

### Ethical approval

All the eight studies were approved by the Hungarian Medical Research Council, details were provided in the referred original manuscripts. Ethical approval for the secondary joint analysis of their data was obtained also from the Hungarian Medical Research Council (no. IV/8070-1/2020/EKU; BM/7790-1/2023).

### Statistics

The joint database was built in Stata 17.0, the exported database was analysed in RStudio 2023.12.1 + 402 “Ocean Storm” Release running on R version 4.3.2. Descriptive statistics were performed.

At first an ordinal logistic regression model was fitted using the ‘polr’ function from the ‘MASS’ package. (‘polr’ stands for Proportional Odds Logistic Regression) Odds ratios of the responses across the levels of the five EQ-5D-5L domains for the two study types were determined. Then the model assumptions were checked by carrying out the Brant test using the R package ’brant’, which suggested that the assumptions did not hold (*p* < 0.0001), i.e., the relationship between each pair of outcome groups was not statistically consistent for the online/personal survey mode partition. Hence, we considered an alternative modelling approach that does not rely on the proportional odds assumption (a.k.a parallel regression assumption). In particular we used multinomial logistic regression, which can model the relationship between the predictors (including mode, age, and sex) and a categorical dependent variable (e.g. Mobility) without assuming proportional odds. In the multinomial logistic regression each level of the outcome variable is treated as a separate category and each category is compared to a reference category. While odds ratios between categories of the outcome variable are not directly calculated, odds ratios of belonging in each category versus the reference category can be calculated for a unit change in the predictors. The p-values associated with the odds ratios indicate the statistical significance of the relationship between the predictor variables and the outcome variable. Specifically, these p-values help to assess whether the observed odds ratios significantly differ from 1, implying whether the predictor has a statistically significant effect on the outcome. The calculation of the p-value for each odds ratio followed standard statistical calculations from Z-values, where the Z-value is the ratio of the estimate to its standard error. The p-value is then calculated using the cumulative distribution function of the standard normal distribution.

The Fisher’s exact test was used to examine whether the distribution of unadjusted scores across the five health domains (Mobility, Self-care, Usual activities, Pain/Discomfort and Anxiety/Depression) differ between the two survey modes. This is a more sophisticated method than the Chi-squared test for contingency tables, however it requires a lot of computational resources. Fisher’s exact test was primarily designed for 2 × 2 tables. For the previous reasons when implementing it in R we rather used the simulated p-value option, which is based on Monte Carlo simulation with 2000 random samples generated from the data.

Linear regression was used to compare EQ-5D-5L index values and EQ VAS scores between the two data collection methods. P-values from Student’s t-tests show whether EQ VAS and on EQ-5D-5L index values differ significantly by survey mode (online vs. CAPI). This was checked across various subgroups as well. In the bivariate cases the models only included the intercept and the survey mode variable (online vs. CAPI), while in the multivariate cases, age and sex variables were also added for adjustment.

To model EQ-5D-5L index values, we applied traditional regression and machine learning regression tools. Linear regression and random forest regression models were fitted using survey mode, education, sex and age as explanatory variables.

## Results

### Joint database

The final database comprised data from 10,324 respondents, of which 7,304 individuals (70.7%) participated in the 6 online surveys and 3,020 individuals (29.3%) in the two CAPI surveys. Sociodemographic characteristics of the sample in the individual studies and in the pooled samples are presented in Table 1, in comparison with the micro census data of Hungary, year 2016 [32].

**Table 1:** Sociodemographic characteristics of the samples in the individual studies and pooled data of the online surveys and CAPI studies

Variables	Total sample		Online surveys (year of data collection)														CAPI studies						Census, 2016
			CarerQol study (2018–2019)		Subjective health expectations (2019)		eHealth literacy study (2019)		MSK-HQ study (2020)		PAM study* (2020)		IMD study* (2021)		Pooled online surveys		Population health study (2019)		ICECAP-A valuation study (2019)		Pooled CAPI studies		
	N	%	N	%	N	%	N	%	N	%	N	%	N	%	N	%	N	%	N	%	N	%	%
**Sample**	10,324	100	1,000	100	1,000	100	1,000	100	2,004	100	900	100	1,400	100	**7**,**304**	100	2,020	100	1,000	100	**3**,**020**	100	
**Gender**																							
male	4,886	47.3	488	48.8	455	45.5	450	45.0	940	46.9	425	47.2	648	46.3	**3**,**406**	**46.6**	1,010	50.0	470	47.0	**1**,**480**	**49.0**	46.9
female	5,438	52.7	512	51.2	545	54.5	550	55.0	1,064	53.1	475	52.8	752	53.7	**3**,**898**	**53.4**	1,010	50.0	530	53.0	**1**,**540**	**51.0**	53.1
**Age group**																							
18–24	706	6.8	39	3.9	52	5.2	118	11.8	192	9.6	NA	NA	NA	NA	**401**	**5.5**	208	10.3	97	9.7	**305**	**10.1**	10.0
25–34	1,187	11.5	97	9.7	115	11.5	198	19.8	306	15.3	NA	NA	NA	NA	**716**	**9.8**	307	15.2	164	16.4	**471**	**15.6**	15.2
35–44*	1,840	17.8	178	17.8	221	22.1	191	19.1	387	19.3	94	10.4	190	13.6	**1**,**261**	**17.3**	386	19.1	193	19.3	**579**	**19.2**	19.5
45–54	1,850	17.9	181	18.1	180	18.0	125	12.5	332	16.6	185	20.6	351	25.1	**1**,**354**	**18.5**	333	16.5	163	16.3	**496**	**16.4**	16.0
55–64	2,104	20.4	227	22.7	192	19.2	147	14.7	328	16.4	276	30.7	425	30.4	**1**,**595**	**21.8**	333	16.5	176	17.6	**509**	**16.9**	16.8
65–74	2,040	19.8	238	23.8	205	20.5	185	18.5	400	20.0	279	31.0	309	22.1	**1**,**616**	**22.1**	267	13.2	157	15.7	**424**	**14.0**	13.0
75+	597	5.8	40	4.0	35	3.5	36	3.6	59	2.9	66	7.3	125	8.9	**361**	**4.9**	186	9.2	50	5.0	**236**	**7.8**	9.5
Education**																							
primary	3,587	34.7	231	23.1	300	30.0	341	34.1	608	30.3	251	27.9	410	29.3	**2**,**141**	**29.3**	931	46.1	515	51.5	**1**,**446**	**47.9**	50.1
secondary	4,066	39.4	374	37.4	422	42.2	363	36.3	968	48.3	326	36.2	533	38.1	**2**,**986**	**40.9**	706	35.0	374	37.4	**1**,**080**	**35.8**	28.7
tertiary	2,671	25.9	395	39.5	278	27.8	296	29.6	428	21.4	323	35.9	457	32.6	**2**,**177**	**29.8**	383	19.0	111	11.1	**494**	**16.4**	21.2
**Type of residence**																							
capital	2,109	20.4	219	21.9	223	22.3	213	21.3	358	17.9	209	23.2	325	22.5	**1**,**537**	**21.0**	399	19.8	173	17.3	**572**	**18.9**	18.1
urban	5,521	53.5	535	53.5	523	52.3	557	55.7	1 053	52.5	513	57.0	749	53.5	**3**,**930**	**53.8**	1 060	52.5	531	53.1	**1**,**591**	**52.7**	52.8
rural	2,694	26.1	246	25.6	254	25.4	230	23.0	593	29.6	178	19.8	336	24.0	**1**,**837**	**25.2**	561	27.8	296	29.6	**857**	**28.4**	29.1

* In the PAM and IMD studies, inclusion criteria was age 40 years and over, hence data refer to age group 40–44 years

**Primary: Did not complete the first year of primary school, Primary school grades 1–3, 4–7 and 8, Vocational qualification without secondary school leaving certificate (e.g., vocational training, vocational school certificate), Vocational qualification obtained within a system based on completion of secondary education; Secondary: Secondary school leaving certificate without vocational qualification, Certificate based on secondary education with vocational qualification (e.g., professional qualification obtained along with secondary school leaving certificate), Certificate obtained in higher education vocational training, No university or college degree; Tertiary: University or college degree

NA = not available

In terms of sex, both the individual study samples and the pooled online and pooled CAPI samples were rather similar to census data. In terms of age, the youngest age groups were underrepresented in two out of the six online studies (ʻCarerQol study’ and ʻSubjective health expectations study’). In addition, only respondents aged 40 years and over were invited to participate in the two online surveys (ʻPAM study’ and ʻIMD study’). The pooled online sample showed underrepresentation of age groups between 18 and 44 years, and overrepresentation of age groups between 45 and 74 years. Age distribution of the pooled CAPI sample was rather similar to census data up to age 74 years. The oldest age group (75 and over) was underrepresented in the pooled online and CAPI samples (4.9% and 7.8%, respectively, versus 9.5%). In terms of educational level, more educated participants were overrepresented in the pooled online sample and underrepresented in the pooled CAPI. Distribution by type of residence in the pooled CAPI was also closer to census data. Overall, the pooled CAPI sample was more similar to the census data (Table 1).

The discrepancies of the single studies compared to census data could have been balanced by weighting the samples to make it representative for the adult Hungarian population by major sociodemographic characteristics. We have checked that the average EQ-5D-5L index values and EQ VAS scores would have differed between the weighted and non-weighted samples by less than 1% (Online Resource 1). Using the original data for comparisons between online and CAPI samples is more appropriate, therefore we decided to omit data weighting for the analyses.

### Results on the EQ-5D-5L descriptive system

In the total sample, the most frequently reported 20 health states on the descriptive system of the EQ-5D-5L are presented in Table 2, in parallel with the respective EQ-5D-5L index values, mean and median EQ VAS scores. ‘No problem’ response option in any of the five domains (ʻ11111’) was reported by the majority (41.2%) of the respondents, followed by reporting ‘slight problems’ only in the pain/discomfort (ʻ11121’; 7.1%) and in the anxiety/depression domain (ʻ11112’; 4.5%). The respective EQ-5D-5L index values varied between 1 and 0.803, and the median EQ VAS of the respondent subgroups was between 90 and 70 (Table 2).

**Table 2:** The twenty most frequently reported health states in the total sample

Total sample, *N* = 10,324						
EQ-5D-5L descriptive system	*N*	%	Cumulative %	EQ-5D-5L index value	Mean EQ VAS	Median EQ VAS
11111	4 251	41.18	41.18	1.000	88.45	90
11121	730	7.07	48.25	0.957	79.81	81
11112	468	4.53	52.78	0.960	82.45	85
11122	423	4.10	56.88	0.917	74.94	80
21121	394	3.82	60.69	0.922	76.32	80
21111	302	2.93	63.62	0.965	82.82	85
21221	186	1.80	65.42	0.887	73.86	74
21122	170	1.65	67.07	0.882	71.88	74
11221	122	1.18	68.25	0.922	74.34	76
21222	108	1.05	69.29	0.847	67.31	70
31221	93	0.90	70.20	0.833	66.30	70
31121	89	0.86	71.06	0.868	73.08	75
11123	81	0.78	71.84	0.864	69.04	73
11113	70	0.68	72.52	0.907	75.23	80
22222	62	0.60	73.12	0.802	64.03	68
11131	60	0.58	73.70	0.927	71.95	75
11211	54	0.52	74.23	0.965	80.24	82
31111	53	0.51	74.74	0.911	77.94	81
11222	51	0.49	75.23	0.882	71.73	73
31231	50	0.48	75.72	0.803	66.20	70

The most frequently reported health states by survey mode are resented in Table 3. In the pooled online sample (*N* = 7,304), the same three health states were the most frequently reported (33.9%, 7.5% and 5.3%, respectively) but with a lower share of perfect health status; the EQ-5D-5L index values were between 1 and 0.802 and the median EQ VAS varied between 90 and 70 (Online Resource 2). In the pooled CAPI sample (*N* = 3,020), again the same three health states were the most common (58.9%, 5.9% and 2.8%, respectively) but with a higher share of perfect health state; the EQ-5D-5L index values were between 1 and 0.802 and the median EQ VAS was between 91 and 60 (Online Resource 3).

**Table 3:** The twenty most frequently reported health states by survey modes. The States in bold are unique

Online		Reported state’s rank	CAPI	
%	EQ-5D-5L descriptive system		EQ-5D-5L descriptive system	%
33.86	11111	1.	11111	58.87
7.54	11121	2.	11121	5.93
5.27	11112	3.	11112	2.75
4.67	11122	4.	11122	2.72
4.30	21121	5.	21121	2.65
3.20	21111	6.	21111	2.25
1.86	21221	7.	21221	1.66
1.74	21122	8.	21122	1.42
1.40	11221	9.	**22222**	0.96
1.19	21222	10.	21222	0.70
1.07	31221	11.	11221	0.66
1.07	**31121**	12.	**22221**	0.66
0.97	**11123**	13.	**21231**	0.60
0.79	11113	14.	**21131**	0.56
0.66	**31111**	15.	11131	0.53
0.63	**11211**	16.	31221	0.50
0.60	11131	17.	**31232**	0.46
0.60	**11222**	18.	**21232**	0.43
0.55	**31231**	19.	**33333**	0.43
0.53	**21211**	20.	11113	0.40

Responses obtained on the five dimensions of the EQ-5D-5L descriptive system are presented in Table 4. Results by age groups, and by age groups for males and females are presented in supplementary files (Online Resource 4, 5 and 6, respectively). In the total sample, most problems were reported in the pain/discomfort domain, followed by mobility, anxiety/depression, usual activities, and self-care domains (46.6%, 34.6%, 29.2%, 25.5% and 11.1%, respectively). In the pooled online and the pooled CAPI samples, the same order of problematic domains was observed but the share across the five domains was different (pooled online: 51.9%, 38.5%, 33.5%, 28.5% and 11.9%, respectively; pooled CAPI: 33.6%, 25.1%, 18.9%, 18.4% and 9.3%, respectively) (Table 4). The difference was statistically significant between the two types of samples in all the five domains, with higher share of respondents indicating no problems in the pooled CAPI sample than in the pooled online sample in all the five domains.

**Table 4:** Distribution of responses on the EQ-5D-5L domains’ level according to pooled samples and comparisons between survey types (online versus CAPI)

	Total		Pooled online surveys		Pooled CAPI studies					
	*N*	%	*N*	%	*N*	%	*p* ^a^	OR	CI	*p*
N	10,324		7,304		3,020					
**Mobility**							< 0.0005			
no	6,756	65.44	4,494	61.53	2,262	74.90				
slight	1,903	18.43	1,449	19.84	454	15.03		1.46	(1.29; 1.65)	***
moderate	1,110	10.75	909	12.45	201	6.66		2.1	(1.78; 2.49)	***
severe	470	4.55	370	5.07	100	3.31		1.78	(1.36; 2.17)	***
unable	85	0.82	82	1.12	3	0.10		12.78	(4.04; 40.54)	***
**Self-care**							< 0.0005			
no	9,177	88.89	6,437	88.13	2,740	90.73				
slight	645	6.25	483	6.61	162	5.36		1.14	(0.95; 1.38)	0.163372172
moderate	347	3.36	261	3.57	86	2.85		1.16	(0.90; 1.47)	0.252603185
severe	105	1.02	74	1.01	31	1.03		0.91	(0.60; 1.39)	0.672806826
unable	50	0.48	49	0.67	1	0.03		20.57	(2.84; 149.30)	0.002781532
**Usual activities**							< 0.0005			
no	7,690	74.49	5,226	71.55	2,464	81.59				
slight	1,622	15.71	1,252	17.14	370	12.25		1.45	(1.28; 1.66)	***
moderate	732	7.09	593	8.12	139	4.60		1.84	(1.51; 2.23)	***
severe	216	2.09	177	2.42	39	1.29		1.94	(1.37; 2.76)	***
unable	64	0.62	56	0.77	8	0.26		3.08	(1.47; 6.48)	0.002979825
**Pain / discomfort**							< 0.0005			
no	5 517	53.44	3 511	48.07	2 006	66.42				
slight	3 158	30.59	2 513	34.41	645	21.36		2.05	(1.85; 2.28)	***
moderate	1 249	12.10	964	13.20	285	9.44		1.77	(1.53; 2.05)	***
severe	307	2.97	230	3.15	77	2.55		1.57	(1.20; 2.04)	0.0009641878
extreme	93	0.90	86	1.18	7	0.23		6.52	(3.01; 14.12)	***
**Anxiety / depression**							< 0.0005			
not	7 311	70.82	4 861	66.55	2 450	81.13				
slightly	2 006	19.43	1 584	21.69	422	13.97		1.88	(1.67; 2.11)	***
moderately	697	6.75	588	8.05	109	3.61		2.69	(2.18; 3.33)	***
severely	219	2.12	188	2.57	31	1.03		3.18	(2.16; 4.67)	***
extremely	91	0.88	83	1.14	8	0.26		5.34	(2.59; 11.15)	***

%: 100% is the column total, ***p < 0.001, p^a^: p-values from Fisher’s exact test, OR: adjusted for age, sex and education, CI: 95% confidence interval for the odds ratios

For the interpretation of Table 4: an odds ratio (OR) of 1.78 for the mode of the survey (online vs. CAPI, with CAPI as the reference) for Mobility level 4 (‘severe’) compared to Mobility level 1 (‘no’) indicates that the odds of a respondent reporting Mobility level 4 (‘severe’) versus Mobility level 1 (‘no’) are 1.78 times higher for those surveyed online compared to those surveyed via CAPI, after adjusting for age and sex. This interpretation assumes a multinomial logistic regression model where Mobility level 1 (ʻno’) serves as the reference category for the outcome. The p-values associated to the odds ratios indicate the statistical significance of the relationship between the predictor variables and the outcome variable. Specifically, these p-values help to assess whether the observed odds ratios significantly differ from 1, implying whether the predictor has a statistically significant effect on the outcome.

### Results of EQ-5D-5L index values and regression analysis

**Fig. 1:**
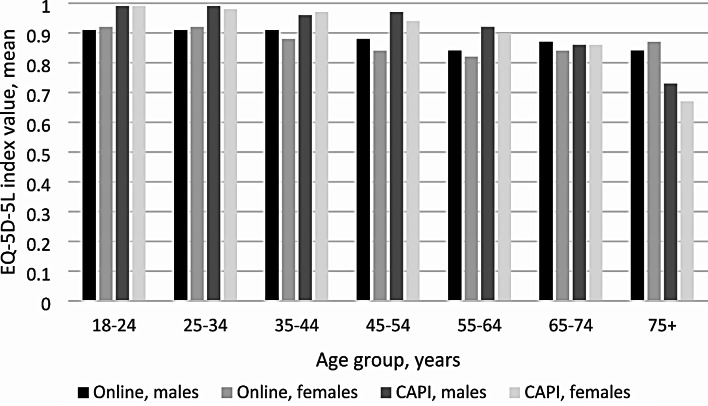
Average EQ-5D-5L index values by sex and age group in the pooled online and pooled CAPI samples

EQ-5D-5L index value results of the total sample, the pooled online and CAPI samples by sociodemographic subgroups are presented in Table 5. (Descriptive statistics separately for males and females by age group and educational level are presented in Online Resource 1.) In the total sample, the average EQ-5D-5L index value was 0.88 (SD = 0.2) and males had a slightly higher average value. A decrease in the average EQ-5D-5L index value by age groups was observed with an outlier increase in age group 65–74. The average EQ-5D-5L index values by age group and sex in the two study types are presented in Fig. 1. In the pooled online sample, the average EQ-5D-5L index value was 0.87 (SD = 0.21) and a similar pattern by age groups was observed as in the total sample. In contrast, the average index value was 0.92 (SD = 0.17) in the pooled CAPI but showed a decrease throughout the age groups. Multivariate regression analysis revealed significantly higher EQ-5D-5L index values in the CAPI than in the online sample. The same was observed in the subgroup analysis by sex and education (Table 5). The subgroup analysis by age revealed significantly higher EQ-5D-5L index values in the CAPI surveys up to age group 55–64, no statistically significant difference was found in age group 65–74, and EQ-5D-5L index values in the CAPI surveys were significantly lower in age group 75 + compared to the online samples (Table 5). However, it should be mentioned, that the sample sizes were quite large, hence relatively small differences could appear as significant ones. Thus, for a deeper insight effect sizes were calculated. The Cohen’s d of 0.28 indicated small effect of the survey modes. It was also calculated for all the subgroups as presented in Table 5. Adjusting for all the explanatory variables standardised mean differences (SMD) were calculated by means of multivariate regression analysis. This yielded 0.29 (small to modest) effect for the survey mode. The other values of SMD-s shown in Table 5. refer to the effect of different levels compared to the reference levels (the latter being online, female, 18–24 and primary for mode, sex, age and education, respectively).

**Table 5:** EQ-5D-5L index values according to sample in different strata, and comparison between modes of survey (online versus CAPI) using bivariate and multivariate linear regression analysis

	Total			Pooled online surveys			Pooled CAPI studies			*p* (online vs. CAPI)		Cohen’s d (Total)	SMD (Total)
	*N*	Mean	SD	*N*	Mean	SD	*N*	Mean	SD	Bivariate	Multivariate*		
**Total**	10,324	0.88	0.2	7,304	0.87	0.21	3,020	0.92	0.17	***	***	0.28	
**Sex**													
male	4,886	0.89	0.2	3,406	0.87	0.21	1,480	0.93	0.16	***	***	0.28	0.11
female	5,438	0.88	0.2	3,898	0.86	0.21	1,540	0.92	0.17	***	***	0.28	
**Age group**													-0.2
18–24	706	0.95	0.14	401	0.91	0.17	305	0.99	0.06	***	***	0.54	
25–34	1,187	0.94	0.12	716	0.92	0.15	471	0.98	0.07	***	***	0.52	
35–44	1,840	0.92	0.18	1,261	0.90	0.2	579	0.97	0.12	***	***	0.39	
45–54	1,850	0.88	0.22	1,354	0.86	0.24	496	0.95	0.11	***	***	0.45	
55–64	2,104	0.85	0.23	1,595	0.83	0.24	509	0.91	0.17	***	***	0.35	
65–74	2,040	0.86	0.19	1,616	0.86	0.19	424	0.86	0.17	0.6924	***	0.02	
75+	597	0.79	0.25	361	0.85	0.17	236	0.70	0.32	***	***	-0.63	
**Education**													0.15
primary	3,587	0.85	0.24	2,141	0.83	0.25	1,446	0.89	0.21	***	***	0.27	
secondary	4,066	0.89	0.19	2,986	0.88	0.2	1,080	0.95	0.13	***	***	0.4	
tertiary	2,671	0.91	0.16	2,177	0.90	0.17	494	0.96	0.1	***	***	0.42	

***p < 0.001

*Adjusted for age and sex

Scale of Cohen’s d and standardised mean difference; small 0.20; medium 0.50; large 0.80

### Results on EQ VAS scores and regression analysis

EQ VAS results are presented in Table 6. (Descriptive statistics separately for males and females by age group and educational level for the total sample and for the two pooled samples are presented in Online Resource 8.) In the total sample, the average EQ VAS score was 77.00 (SD = 19.55), males had slightly higher average score than females, a decrease by increasing age was observed and respondents with primary education had lower average score than respondents with secondary or tertiary education. Bivariate and multivariate regression analysis was performed to compare EQ VAS scores in the online and CAPI pooled samples (as described above at the EQ-5D-5L index value analyses). EQ VAS scores were significantly higher in the pooled CAPI sample in all subgroups by sex and education level. The subgroup analysis by age group showed a similar pattern to the EQ-5D-5L index values, as EQ VAS scores were also significantly higher in the pooled CAPI sample up to age 64, no significant difference was observed in the age group 65–74 and significantly higher scores were found in the online sample in the oldest age group (Table 6). The same remarks regarding significance apply here as well as for the EQ-5D-5L index. Both the unadjusted (Cohen’s d) and adjusted (SMD) effect size is 0.31 for the survey mode, which is considered as small to modest, again.

**Table 6:** EQ VAS results according to sample in different strata, and comparison between modes of survey (online versus CAPI) using bivariate and multivariate linear regression analysis

	Total			Pooled online surveys			Pooled CAPI studies			*p* (online vs. CAPI)		Cohen’s d (Total)	SMD (Total)
	N	Mean	SD	N	Mean	SD	N	Mean	SD	Bivariate	Multivariate*		
**Total**	10,324	77.00	19.55	7,304	75.22	19.91	3,020	81.32	17.95	***	***	0.31	
**Sex**													
male	4,886	77.13	19.38	3,406	75.00	19.72	1,480	82.03	17.62	***	***	0.31	0.06
female	5,438	76.89	19.71	3,898	75.41	20.08	1,540	80.64	18.24	***	***	0.31	
**Age group**													-0.25
18–24	706	86.26	15.93	401	80.99	16.99	305	93.20	11.11	***	***	0.83	
25–34	1,187	83.74	17.40	716	79.15	18.89	471	90.71	11.84	***	***	0.7	
35–44	1,840	79.96	19.04	1,261	76.88	20.42	579	86.65	13.36	***	***	0.53	
45–54	1,850	78.13	19.13	1,354	75.90	20.01	496	84.21	14.92	***	***	0.44	
55–64	2,104	73.57	20.12	1,595	72.86	20.77	509	75.82	17.76	0.00373	***	0.15	
65–74	2,040	72.51	19.12	1,616	73.22	19.40	424	69.83	17.77	0.00116	0.190273	-0.18	
75+	597	67.52	19.11	361	72.09	17.84	236	60.54	18.90	***	0.00995	-0.63	
**Education**													0.15
primary	3,587	73.79	21.48	2,141	71.79	22.25	1,446	76.73	19.95	***	***	0.23	
secondary	4,066	78.67	18.79	2,986	76.35	19.37	1,080	85.08	15.36	***	***	0.47	
tertiary	2,671	78.79	17.31	2,177	77.04	17.67	494	86.50	13.07	***	***	0.56	

***p < 0.001

*Adjusted for age and sex

Scale of Cohen’s d and standardised mean difference; small 0.20; medium 0.50; large 0.80

### Modelling the determinants of EQ-5D-5L index values

Considering survey mode, education, sex and age as explanatory variables, we got 7–10% of variance explained in the data. This shows that the models have not much predictive power, however both analyses yielded that age seems to have the largest effect on EQ-5D-5L index values and sex seems to have the lowest (data not shown).

## Discussion

We pooled together six online and two CAPI cross-sectional population-based surveys in Hungary that involved EQ-5D-5L results, to provide country-specific EQ-5D-5L population norms and to investigate the differences between the two survey modes. We have built a joint database (*N* = 10,324) comprising basic sociodemographic characteristics (age, sex, education), EQ-5D-5L descriptive system results, index values and EQ VAS data. The two survey modes showed similar trends in certain aspects, as the EQ-5D-5L index and EQ VAS scores were significantly higher among men than in women, and higher scores were seen in subgroups with higher educational levels. ‘No problem’ in any of the five EQ-5D-5L domains was reported by most of the respondents in both the online and CAPI studies but it was more frequent in the latter (33.9% vs. 58.9%, respectively). Most problems were reported in the pain/discomfort domain in both survey modes although at different frequency levels (online: 51.9%; CAPI: 33.6% of respondents reported any problems). Multivariate regression analysis revealed that EQ-5D-5L index values and EQ VAS scores were significantly higher in the pooled CAPI sample compared to the pooled online sample. The same was observed in subgroup analyses by sex and by education. However, subgroup analyses by age groups showed significantly higher scores up to age 64 for both measures. No significant difference was observed in age group 65–74, but in the highest age group (age 75 and over) EQ-5D-5L index values and EQ VAS scores were significantly higher in the online sample. Considering survey mode, education, sex and age, only 7–10% of variance in the EQ-5D-5L index values was explained, with age having the largest and sex the smallest effect.

The significant difference in EQ-5D-5L index values and EQ VAS scores between the online and CAPI samples raises the question which one to use as population norms for Hungary. Sociodemographic characteristics of the CAPI sample were closer to the Hungarian population census data; therefore, the CAPI population norms seem to be more appropriate. Recruitment for the online samples was performed among members of online survey panels who have internet access, show online activity and are willing to participate in surveys, hence they may differ from the average population due to this selection bias. We found, indeed, that more educated people were overrepresented in the online samples. Nonetheless, the online population norms may also be relevant with the digitalization of healthcare, more and more data collections are conducted via (or from) the internet and an increasing number of patient studies rely on online available individuals [33–36]. Their results are often considered as representative for the ʻonline population’ hence the online population norms can be a useful reference set in these cases, particularly in specific subgroups, such as the elderly.

Although we have not formulated preliminary hypotheses, we subjectively expected that individuals being active online and participating in online survey panels were, in average, in a better health status and thus would provide higher EQ-5D-5L index values and EQ VAS scores compared to the CAPI sample. However, we have found the opposite, except for age groups 65–74 (no significant difference) and 75 (higher scores in the online sample). We assume that it is not the EQ-5D-5L administration mode itself that induced the difference between the two survey types. In the online surveys, the self-completed digital version of the EQ-5D-5L was applied and no interviewer was present, which is indeed a major difference compared to the CAPI studies. In one of the two CAPI studies (the ʻPopulation health study’) [28–30], the paper-based self-completed version was filled in by the participants (but in the presence of the interviewer). In the other CAPI study (ʻICECAP-A valuation study’) [31], the self-completed digital version was used, and interviewers were trained to turn the computer screen to the respondents and let them fill in this part by themselves. We assume that the differences between the online and CAPI studies were rather originated from the difference in settings (online panel versus residents living in their home) and recruitment methods (invitation by email vs. random walk). We know only about one study (Jiang and colleagues, USA) that compared EQ-5D-5L population norms obtained from different studies in the same country, i.e., from a face-to-face study (*N* = 1,134) and from an online survey (*N* = 2,018) [12]. Similarly to our research, lower values were found in the online sample than in the interview study. As Jiang and colleagues highlighted, the face-to-face interviews were performed centralized in their study thus participants had to be able to travel or being transported. Authors also acknowledged the potential impact of other factors such as the difference in the representation of minorities. Our data does not allow further in-depth analyses of the determinants of the differences. Overall, we think that our results reflect partly real differences between the health status of the two samples, but we cannot exclude the impact of the interviewer being present in the CAPI design. In addition, the topic, the content, the length of the survey questionnaire itself and the position of the EQ-5D-5L within the survey might have an effect on the responses, but other potential confounding factors also deserve investigation in further studies.

The EQ-5D-5L population norms study by Nikl and colleagues in Hungary was based on an online survey (*N* = 1,631) in which, similarly to our online studies, recruitment was performed by a panel company and quotas were set in terms of age, sex, education, place of residence and geographic region [13]. The most commonly reported problem was pain/discomfort just as in our pooled online sample. Average EQ-5D-5L index values by age groups showed very small differences compared to our pooled online data. Overall, their study results are in line with our online results, but our findings with respect to differences between online and CAPI studies also highlight the limitations of online based population norms.

We have conducted a rapid review in PubMed for EQ-5D-5L population norms publications to have an insight into the previous studies on the international level. Acknowledging that we may have missed some relevant articles, we have identified 41 publications reporting results from 36 geographic locations. Detailed analysis of these studies would go beyond the scope of our current research. In brief though, the great majority were completed in interview situations although using different modes of administration of the EQ-5D-5L (*N* = 26; Australia – South [37], Barbados [38], Belgium [39], Belize [40], Bulgaria [41], China [42], China – urban Chinese [43], China – Jiangsu province [44], Canada-Alberta and Quebec [45], Colombia [46], Denmark [47], Germany [9, 48], Hong Kong [49], India [50], Indonesia [51], Ireland [52], Jamaica [38], Japan [53], Moscow [54], Poland [55], Romania [10], Spain [56, 57], Thailand [58], Trinidad and Tobago [59], USA [12], Vietnam [60]). There were much less online studies (*N* = 9, Australia [61], Canada-Alberta [62], Canada-Quebec [63], France [64], Hungary [13], Netherlands-females [65], New Zealand [66], Slovenia [67], USA [12]), the other studies were phone interviews (*N* = 3; Australia-Queensland [68], Germany [69], Portugal [70]), postal surveys (*N* = 2; Norway [71], Sweden [72]) and an online video survey (*N* = 1; Italy [73]), indicating that several approaches have been used worldwide to obtain EQ-5D-5L population norms. Results from different survey modes in one country (although from somewhat different regions and in years) were reported only from Australia (interview, online, phone interview) [37, 61, 68], Canada (interview, online) [45, 62, 63], the USA (interview, online) [12], Germany (CAPI, phone interview). However, comparisons between two studies were performed only in the USA, as we discussed above [12]. Regarding the sample size, some studies included large samples and these were not proportional across the studies in terms of the size of the population of the given country (e.g., postal survey: Sweden *N* = 25,867; phone interview: Australia-Queensland *N* = 25,170; interviews: Spain *N* = 21,007; China-Jiangsu Province *N* = 10,056; Japan *N* = 10,183; Canada-Alberta *N* = 8,790 and *N* = 9,263; Belgium *N* = 7,509; online survey: France: *N* = 15,000; Netherland *N* = 9,037). Nonetheless, most samples were much smaller (mainly under 3000 participants) and the EQ-5D-5L valuation studies involving around 1000 individuals were also used to establish population norms. In the light of these results, we think that an important strength of our research is that our database is among the larger ones, comprising multiple databases and different survey modes from one country.

Regarding population norms from our geographic region, the share of participants reporting no problem (ʻ11111’) in any of the five EQ-5D-5L domains was 50.1% in Bulgaria (BG; omnibus survey, paper) [41], 38.5% in Poland (PL; interview, paper) [55], 50.3% in Romania (RO; interview, digital) [10] and 28.2% in Slovenia (SI; online, digital) [67], while it was 58.9% and 33.9% in our CAPI and online samples, respectively. In all the four neighbouring countries most problems were reported in the pain/discomfort domain (BG: 39.1%, PL: 52.2%, RO: 39.9%, SI: 58.1%) followed by the anxiety/depression domain (BG: 34.5%; PL: 41.5%; RO: 34.5%, SI: 38.9%). While pain/discomfort was also the most problematic domain in our study (online: 51.9%; CAPI: 33.6%), the second most reported problems were in the mobility domain (online: 38.5%, CAPI: 25.1%). These differences highlight the importance of developing country-specific population norms within a geographic region.

It is important to acknowledge the limitations of our study and, in this regard, highlight some directions for further research. First, we used previously collected datasets to compare online and CAPI surveys, hence the data allowed only indirect comparisons. Second, the six online studies recruited participants from large online panels via market survey companies between 2018 and 2021. Details of this type of recruitment are confidential business information, making this part of the studies poorly transparent. We cannot completely exclude overlap between the six online study samples. However, analyses on the study level showed differences in terms of sociodemographic characteristics hence we assume that the coincidence was, if any, insignificant. This potential bias could have been eliminated if only one online study was compared to one CAPI study, but we decided to use larger joint datasets to strengthen the power of the analyses. Third, as we used previously collected data, we could not investigate the reasons for the differences between the results of the online and CAPI studies, as we discussed above. Fourth, we considered only basic sociodemographic characteristics of the respondents (age, sex, education). Although these three variables are the most frequently used to establish EQ-5D-5L population norms, we believe that further individual characteristics and contextual factors may have significant impact on EQ-5D-5L results and deserve consideration in future studies. Fifth, we relied on cross-sectional surveys, therefore we could not investigate the differences between survey modes regarding responsiveness to changes. Given that measuring changes in health status of the population is one of the most relevant applications of health status measurement tools in public health, we believe examining this aspect should be a priority in future research.

In conclusion, our research findings suggest that both EQ-5D-5L and EQ VAS population norms originated from online studies and from CAPI studies may differ significantly from each other. In base case, we suggest using the CAPI population norms for Hungary. The online EQ-5D-5L population norms deserve consideration as reference set for disease burden and other health studies that involve samples representative for the online available population. Given the scarcity of comparative studies investigating EQ-5D-5L population norms by different study designs and administration modes, we encourage further research to this end.

## Electronic supplementary material

Below is the link to the electronic supplementary material.

Supplementary Material 1

Supplementary Material 2

Supplementary Material 3

Supplementary Material 4

Supplementary Material 5

Supplementary Material 6

Supplementary Material 7

Supplementary Material 8

## Data Availability

Database of this study is available from the corresponding author upon reasonable request.
