# Supplementary material for: Population norms for the EQ-5D-5L for Hungary: comparison of online surveys and computer assisted personal interviews
Source: Eur J Health Econ. 2025 Feb 21;26(6):1111–26. doi: 10.1007/s10198-024-01755-2 (PMC12310892; doi:10.1007/s10198-024-01755-2)
Supplement: Supplementary file 1 — Supplementary Material 1 [file 10198_2024_1755_MOESM1_ESM.docx]

Online Resource 1 Mean EQ-5D-5L index values and EQ VAS scores in the not weighted and weighted samples

|  | Total sample | Online | CAPI |
| --- | --- | --- | --- |
| EQ-5D-5L index value, mean |  |  |  |
| non-weighted | 0.884 | 0.867 | 0.923 |
| weighted | 0.878 | 0.863 | 0.919 |
| EQ VAS, mean |  |  |  |
| not weighted | 77.00 | 75.22 | 81.32 |
| weighted | 76.92 | 75.10 | 81.20 |
