# Supplementary material for: Population norms for the EQ-5D-5L for Hungary: comparison of online surveys and computer assisted personal interviews
Source: Eur J Health Econ. 2025 Feb 21;26(6):1111–26. doi: 10.1007/s10198-024-01755-2 (PMC12310892; doi:10.1007/s10198-024-01755-2)
Supplement: Supplementary file 2 — Supplementary Material 2 [file 10198_2024_1755_MOESM2_ESM.docx]

Online Resource2 The most frequently reported EQ-5D-5L health states in the pooled online sample

| Online | | | | | | |
| --- | --- | --- | --- | --- | --- | --- |
| N=7 304 | | | | | | |
| eq5p | N | % | Cumulative % | EQ-5D-5L index value | Mean EQ VAS | Median EQ VAS |
| 11111 | 2 473 | 33.86 | 33.86 | 1 | 87.04 | 90 |
| 11121 | 551 | 7.54 | 41.40 | 0.957000017 | 79.66 | 82 |
| 11112 | 385 | 5.27 | 46.67 | 0.959999979 | 82.09 | 85 |
| 11122 | 341 | 4.67 | 51.34 | 0.916999996 | 75.37 | 80 |
| 21121 | 314 | 4.30 | 55.64 | 0.921999991 | 77.11 | 80 |
| 21111 | 234 | 3.20 | 58.84 | 0.964999974 | 83.78 | 85 |
| 21221 | 136 | 1.86 | 60.71 | 0.887000024 | 75.35 | 80 |
| 21122 | 127 | 1.74 | 62.45 | 0.882000029 | 71.99 | 75 |
| 11221 | 102 | 1.40 | 63.84 | 0.921999991 | 75.58 | 78.50 |
| 21222 | 87 | 1.19 | 65.03 | 0.847000003 | 67.77 | 71 |
| 31221 | 78 | 1.07 | 66.10 | 0.833000004 | 67.63 | 71 |
| 31121 | 78 | 1.07 | 67.17 | 0.867999971 | 72.76 | 74 |
| 11123 | 71 | 0.97 | 68.14 | 0.864000022 | 69.54 | 73 |
| 11113 | 58 | 0.79 | 68.93 | 0.907000005 | 75.02 | 80 |
| 22222 | 33 | 0.45 | 69.39 | 0.801999986 | 64.12 | 70 |
| 11131 | 44 | 0,60 | 69.99 | 0.926999986 | 71.89 | 73 |
| 11211 | 46 | 0.63 | 70.62 | 0.964999974 | 80.11 | 82 |
| 31111 | 48 | 0.66 | 71.28 | 0.911000013 | 78.77 | 81 |
| 11222 | 44 | 0.60 | 71.88 | 0.882000029 | 71.89 | 72 |
| 31231 | 40 | 0.55 | 72.43 | 0.802999973 | 66.63 | 70.50 |
