# Supplementary material for: Population norms for the EQ-5D-5L for Hungary: comparison of online surveys and computer assisted personal interviews
Source: Eur J Health Econ. 2025 Feb 21;26(6):1111–26. doi: 10.1007/s10198-024-01755-2 (PMC12310892; doi:10.1007/s10198-024-01755-2)
Supplement: Supplementary file 3 — Supplementary Material 3 [file 10198_2024_1755_MOESM3_ESM.docx]

Online Resource 3 The most frequently reported EQ-5D-5L health states in the pooled CAPI sample

| CAPI | | | | | | |
| --- | --- | --- | --- | --- | --- | --- |
| N=3 020 | | | | | | |
| eq5p | N | % | Cumulative % | EQ-5D-5L index value | Mean EQ VAS | Median EQ VAS |
| 11111 | 1 778 | 58.87 | 58.87 | 1 | 90.41 | 91 |
| 11121 | 179 | 5.93 | 64.80 | 0.957000017 | 80.28 | 80 |
| 11112 | 83 | 2.75 | 67.55 | 0.959999979 | 84.08 | 90 |
| 11122 | 82 | 2.72 | 70.26 | 0.916999996 | 73.15 | 80 |
| 21121 | 80 | 2.65 | 72.91 | 0.921999991 | 73.20 | 73.50 |
| 21111 | 68 | 2.25 | 75.17 | 0.964999974 | 79.53 | 81 |
| 21221 | 50 | 1.66 | 76.82 | 0.887000024 | 69.82 | 70 |
| 21122 | 43 | 1.42 | 78.25 | 0.882000029 | 71.56 | 70 |
| 11221 | 20 | 0.66 | 78.91 | 0.921999991 | 68.05 | 70 |
| 21222 | 21 | 0.70 | 79.60 | 0.847000003 | 65.43 | 65 |
| 31221 | 15 | 0.50 | 80.10 | 0.833000004 | 59.40 | 60 |
| 31121 | 11 | 0.36 | 80.46 | 0.867999971 | 75.36 | 75 |
| 11123 | 10 | 0.33 | 80.79 | 0.864000022 | 65.50 | 70 |
| 11113 | 12 | 0.40 | 81.19 | 0.907000005 | 76.25 | 82.50 |
| 22222 | 29 | 0.96 | 82.15 | 0.801999986 | 63.93 | 65 |
| 11131 | 16 | 0.53 | 82.68 | 0.926999986 | 72.13 | 80 |
| 11211 | 8 | 0.26 | 82.95 | 0.964999974 | 81.00 | 82.50 |
| 31111 | 5 | 0.17 | 83.11 | 0.911000013 | 70.00 | 70 |
| 11222 | 7 | 0.23 | 83.34 | 0.882000029 | 70.71 | 75 |
| 31231 | 10 | 0.33 | 83.68 | 0.802999973 | 64.50 | 65 |
