# Supplementary material for: Population norms for the EQ-5D-5L for Hungary: comparison of online surveys and computer assisted personal interviews
Source: Eur J Health Econ. 2025 Feb 21;26(6):1111–26. doi: 10.1007/s10198-024-01755-2 (PMC12310892; doi:10.1007/s10198-024-01755-2)
Supplement: Supplementary file 4 — Supplementary Material 4 [file 10198_2024_1755_MOESM4_ESM.docx]

Online Resource 4 Distribution of responses on the descriptive system of the EQ-5D-5L by age groups in the total sample

| Total sample, age group, years | | | | | | | | | | | | | | |
| --- | --- | --- | --- | --- | --- | --- | --- | --- | --- | --- | --- | --- | --- | --- |
|  | 18-24 | | 25-34 | | 35-44 | | 45-54 | | 55-64 | | 65-74 | | 75+ | |
|  | N | % | N | % | N | % | N | % | N | % | N | % | N | % |
| N | 706 |  | 1 187 |  | 1 840 |  | 1 850 |  | 2 104 |  | 2 040 |  | 597 |  |
| **Mobility** |  |  |  |  |  |  |  |  |  |  |  |  |  |  |
| no | 641 | 90.79 | 1 057 | 89.05 | 1 486 | 80.76 | 1 300 | 70.27 | 1 154 | 54.85 | 940 | 46.08 | 178 | 29.82 |
| slight | 45 | 6.37 | 93 | 7.83 | 209 | 11.36 | 317 | 17.14 | 478 | 22.72 | 583 | 28.58 | 178 | 29.82 |
| moderate | 14 | 1.98 | 23 | 1.94 | 94 | 5.11 | 149 | 8.05 | 304 | 14.45 | 371 | 18.19 | 155 | 25.96 |
| severe | 5 | 0.71 | 9 | 0.76 | 32 | 1.74 | 58 | 3.14 | 148 | 7.03 | 134 | 6.57 | 84 | 14.07 |
| unable | 1 | 0.14 | 5 | 0.42 | 19 | 1.03 | 26 | 1.41 | 20 | 0.95 | 12 | 0.59 | 2 | 0.34 |
| **Self-care** |  |  |  |  |  |  |  |  |  |  |  |  |  |  |
| no | 679 | 96.18 | 1 149 | 96.80 | 1 722 | 93.59 | 1 693 | 91.51 | 1 781 | 84.65 | 1 723 | 84.46 | 430 | 72.03 |
| slight | 18 | 2.55 | 24 | 2.02 | 66 | 3.59 | 77 | 4.16 | 169 | 8.03 | 200 | 9.80 | 91 | 15.24 |
| moderate | 7 | 0.99 | 8 | 0.67 | 32 | 1.74 | 42 | 2.27 | 106 | 5.04 | 98 | 4.80 | 54 | 9.05 |
| severe | 0 | 0.00 | 5 | 0.42 | 9 | 0.49 | 22 | 1.19 | 32 | 1.52 | 16 | 0.78 | 21 | 3.52 |
| unable | 2 | 0.28 | 1 | 0.08 | 11 | 0.60 | 16 | 0.86 | 16 | 0.76 | 3 | 0.15 | 1 | 0.17 |
| **Usual activities** |  |  |  |  |  |  |  |  |  |  |  |  |  |  |
| no | 640 | 90.65 | 1 071 | 90.23 | 1 583 | 86.03 | 1 427 | 77.14 | 1 413 | 67.16 | 1 268 | 62.16 | 288 | 48.24 |
| slight | 47 | 6.66 | 81 | 6.82 | 171 | 9.29 | 271 | 14.65 | 378 | 17.97 | 486 | 23.82 | 188 | 31.49 |
| moderate | 12 | 1.70 | 24 | 2.02 | 53 | 2.88 | 100 | 5.41 | 226 | 10.74 | 227 | 11.13 | 90 | 15.08 |
| severe | 4 | 0.57 | 8 | 0.67 | 22 | 1.20 | 36 | 1.95 | 70 | 3.33 | 48 | 2.35 | 28 | 4.69 |
| unable | 3 | 0.42 | 3 | 0.25 | 11 | 0.60 | 16 | 0.86 | 17 | 0.81 | 11 | 0.54 | 3 | 0.50 |
| **Pain/discomfort** |  |  |  |  |  |  |  |  |  |  |  |  |  |  |
| no | 538 | 76.20 | 852 | 71.78 | 1 205 | 65.49 | 1 019 | 55.08 | 937 | 44.53 | 791 | 38.77 | 175 | 29.31 |
| slight | 114 | 16.15 | 253 | 21.31 | 462 | 25.11 | 560 | 30.27 | 727 | 34.55 | 792 | 38.82 | 250 | 41.88 |
| moderate | 40 | 5.67 | 68 | 5.73 | 122 | 6.63 | 186 | 10.05 | 322 | 15.30 | 384 | 18.82 | 127 | 21.27 |
| severe | 9 | 1.27 | 12 | 1.01 | 37 | 2.01 | 61 | 3.30 | 86 | 4.09 | 62 | 3.04 | 40 | 6.70 |
| extreme | 5 | 0.71 | 2 | 0.17 | 14 | 0.76 | 24 | 1.30 | 32 | 1.52 | 11 | 0.54 | 5 | 0.84 |
| **Anxiety/depression** |  |  |  |  |  |  |  |  |  |  |  |  |  |  |
| no | 532 | 75.35 | 841 | 70.85 | 1 299 | 70.60 | 1 281 | 69.24 | 1 450 | 68.92 | 1 487 | 72.89 | 421 | 70.52 |
| slight | 107 | 15.16 | 241 | 20.30 | 370 | 20.11 | 350 | 18.92 | 423 | 20.10 | 393 | 19.26 | 122 | 20.44 |
| moderate | 43 | 6.09 | 76 | 6.40 | 113 | 6.14 | 133 | 7.19 | 161 | 7.65 | 131 | 6.42 | 40 | 6.70 |
| severe | 18 | 2.55 | 22 | 1.85 | 35 | 1.90 | 64 | 3.46 | 51 | 2.42 | 17 | 0.83 | 12 | 2.01 |
| extreme | 6 | 0.85 | 7 | 0.59 | 23 | 1.25 | 22 | 1.19 | 19 | 0.90 | 12 | 0.59 | 2 | 0.34 |
