# Supplementary material for: Population norms for the EQ-5D-5L for Hungary: comparison of online surveys and computer assisted personal interviews
Source: Eur J Health Econ. 2025 Feb 21;26(6):1111–26. doi: 10.1007/s10198-024-01755-2 (PMC12310892; doi:10.1007/s10198-024-01755-2)
Supplement: Supplementary file 5 — Supplementary Material 5 [file 10198_2024_1755_MOESM5_ESM.docx]

Online Resource 5 Distribution of responses on the EQ-5D-5L descriptive system by age groups among males in the total sample

| Male only, age group, years | | | | | | | | | | | | | | |
| --- | --- | --- | --- | --- | --- | --- | --- | --- | --- | --- | --- | --- | --- | --- |
|  | 18-24 | | 25-34 | | 35-44 | | 45-54 | | 55-64 | | 65-74 | | 75+ | |
|  | N | % | N | % | N | % | N | % | N | % | N | % | N | % |
| N | 248 |  | 463 |  | 881 |  | 899 |  | 976 |  | 1 098 |  | 321 |  |
| **Mobility** |  |  |  |  |  |  |  |  |  |  |  |  |  |  |
| no | 233 | 93.95 | 414 | 89.42 | 727 | 82.52 | 675 | 75.08 | 558 | 57.17 | 537 | 48.91 | 98 | 30.53 |
| slight | 8 | 3.23 | 31 | 6.70 | 95 | 10.78 | 133 | 14.79 | 204 | 20.90 | 314 | 28.60 | 98 | 30.53 |
| moderate | 4 | 1.61 | 11 | 2.38 | 37 | 4.20 | 58 | 6.45 | 132 | 13.52 | 170 | 15.48 | 84 | 26.17 |
| severe | 3 | 1.21 | 4 | 0.86 | 12 | 1.36 | 28 | 3.11 | 78 | 7.99 | 72 | 6.56 | 41 | 12.77 |
| unable | 0 | 0.00 | 3 | 0.65 | 10 | 1.14 | 5 | 0.56 | 4 | 0.41 | 5 | 0.46 | 0 | 0.00 |
| **Self-care** |  |  |  |  |  |  |  |  |  |  |  |  |  |  |
| no | 235 | 94.76 | 442 | 95.46 | 823 | 93.42 | 829 | 92.21 | 836 | 85.66 | 918 | 83.61 | 224 | 69.78 |
| slight | 9 | 3.63 | 12 | 2.59 | 33 | 3.75 | 27 | 3.00 | 69 | 7.07 | 120 | 10.93 | 53 | 16.51 |
| moderate | 4 | 1.61 | 5 | 1.08 | 17 | 1.93 | 23 | 2.56 | 49 | 5.02 | 52 | 4.74 | 33 | 10.28 |
| severe | 0 | 0.00 | 3 | 0.65 | 4 | 0.45 | 13 | 1.45 | 17 | 1.74 | 7 | 0.64 | 10 | 3.12 |
| unable | 0 | 0.00 | 1 | 0.22 | 4 | 0.45 | 7 | 0.78 | 5 | 0.51 | 1 | .,09 | 1 | 0.31 |
| **Usual activities** |  |  |  |  |  |  |  |  |  |  |  |  |  |  |
| no | 224 | 90.32 | 413 | 89.20 | 770 | 87.40 | 737 | 81.98 | 672 | 68.85 | 702 | 63.93 | 156 | 48.60 |
| slight | 17 | 6.85 | 32 | 6.91 | 77 | 8.74 | 97 | 10.79 | 163 | 16.70 | 253 | 23.04 | 102 | 31.78 |
| moderate | 3 | 1.21 | 15 | 3.24 | 19 | 2.16 | 36 | 4.00 | 103 | 10.55 | 115 | 10.47 | 46 | 14.33 |
| severe | 4 | 1.61 | 3 | 0.65 | 10 | 1.14 | 21 | 2.34 | 33 | 3.38 | 23 | 2.09 | 15 | 4.67 |
| unable | 0 | 0.00 | 0 | 0.00 | 5 | 0.57 | 8 | 0.89 | 5 | 0.51 | 5 | 0.46 | 2 | 0.62 |
| **Pain/discomfort** |  |  |  |  |  |  |  |  |  |  |  |  |  |  |
| no | 208 | 83.87 | 361 | 77.97 | 615 | 69.81 | 555 | 61.74 | 462 | 47.34 | 468 | 42.62 | 98 | 30.53 |
| slight | 25 | 10.08 | 78 | 16.85 | 201 | 22.81 | 238 | 26.47 | 319 | 32.68 | 405 | 36.89 | 143 | 44.55 |
| moderate | 9 | 3.63 | 19 | 4.10 | 47 | 5.33 | 69 | 7.68 | 147 | 15.06 | 192 | 17.49 | 62 | 19.31 |
| severe | 5 | 2.02 | 3 | 0.65 | 13 | 1.48 | 27 | 3.00 | 34 | 3.48 | 29 | 2.64 | 17 | 5.30 |
| extreme | 1 | 0.40 | 2 | 0.43 | 5 | 0.57 | 10 | 1.11 | 14 | 1.43 | 4 | 0.36 | 1 | 0.31 |
| **Anxiety/depression** |  |  |  |  |  |  |  |  |  |  |  |  |  |  |
| no | 202 | 81.45 | 363 | 78.40 | 642 | 72.87 | 673 | 74.86 | 717 | 73.46 | 862 | 78.51 | 241 | 75.08 |
| slight | 26 | 10.48 | 68 | 14.69 | 166 | 18.84 | 143 | 15.91 | 169 | 17.32 | 168 | 15.30 | 56 | 17.45 |
| moderate | 12 | 4.84 | 21 | 4.54 | 52 | 5.90 | 43 | 4.78 | 66 | 6.76 | 55 | 5.01 | 19 | 5.92 |
| severe | 5 | 2.02 | 8 | 1.73 | 11 | 1.25 | 33 | 3.67 | 20 | 2.05 | 11 | 1.00 | 4 | 1.25 |
| extreme | 3 | 1.21 | 3 | 0.65 | 10 | 1.14 | 7 | 0.78 | 4 | 0.41 | 2 | 0.18 | 1 | 0.31 |
