# Supplementary material for: Population norms for the EQ-5D-5L for Hungary: comparison of online surveys and computer assisted personal interviews
Source: Eur J Health Econ. 2025 Feb 21;26(6):1111–26. doi: 10.1007/s10198-024-01755-2 (PMC12310892; doi:10.1007/s10198-024-01755-2)
Supplement: Supplementary file 6 — Supplementary Material 6 [file 10198_2024_1755_MOESM6_ESM.docx]

Online Resource 6 Distribution of responses on the EQ-5D-5L descriptive system by age groups among females in the total sample

| Female only, age group, years | | | | | | | | | | | | | | |
| --- | --- | --- | --- | --- | --- | --- | --- | --- | --- | --- | --- | --- | --- | --- |
|  | 18-24 | | 25-34 | | 35-44 | | 45-54 | | 55-64 | | 65-74 | | 75+ | |
|  | N | % | N | % | N | % | N | % | N | % | N | % | N | % |
| N | 458 |  | 724 |  | 959 |  | 951 |  | 1 128 |  | 942 |  | 276 |  |
| **Mobility** |  |  |  |  |  |  |  |  |  |  |  |  |  |  |
| no | 408 | 89.08 | 643 | 88.81 | 759 | 79.14 | 625 | 65.72 | 596 | 52.84 | 403 | 42.78 | 80 | 28.99 |
| slight | 37 | 8.08 | 62 | 8.56 | 114 | 11.89 | 184 | 19.35 | 274 | 24.29 | 269 | 28.56 | 80 | 28.99 |
| moderate | 10 | 2.18 | 12 | 1.66 | 57 | 5.94 | 91 | 9.57 | 172 | 15.25 | 201 | 21.34 | 71 | 25.72 |
| severe | 2 | 0.44 | 5 | 0.69 | 20 | 2.09 | 30 | 3.15 | 70 | 6.21 | 62 | 6.58 | 43 | 15.58 |
| unable | 1 | 0.22 | 2 | 0.28 | 9 | 0.94 | 21 | 2.21 | 16 | 1.42 | 7 | 0.74 | 2 | 0.72 |
| **Self-care** |  |  |  |  |  |  |  |  |  |  |  |  |  |  |
| no | 444 | 96.94 | 707 | 97.65 | 899 | 93.74 | 864 | 90.85 | 945 | 83.78 | 805 | 85.46 | 206 | 74.64 |
| slight | 9 | 1.97 | 12 | 1.66 | 33 | 3.44 | 50 | 5.26 | 100 | 8.87 | 80 | 8.49 | 38 | 13.77 |
| moderate | 3 | 0.66 | 3 | 0.41 | 15 | 1.56 | 19 | 2.00 | 57 | 5.05 | 46 | 4.88 | 21 | 7.61 |
| severe | 0 | 0.00 | 2 | 0.28 | 5 | 0.52 | 9 | 0.95 | 15 | 1.33 | 9 | 0.96 | 11 | 3.99 |
| unable | 2 | 0.44 | 0 | 0.00 | 7 | 0.73 | 9 | 0.95 | 11 | 0.98 | 2 | 0.21 | 0 | 0.00 |
| **Usual activities** |  |  |  |  |  |  |  |  |  |  |  |  |  |  |
| no | 416 | 90.83 | 658 | 90.88 | 813 | 84.78 | 690 | 72.56 | 741 | 65.69 | 566 | 60.08 | 132 | 47.83 |
| slight | 30 | 6.55 | 49 | 6.77 | 94 | 9.80 | 174 | 18.30 | 215 | 19.06 | 233 | 24.73 | 86 | 31.16 |
| moderate | 9 | 1.97 | 9 | 1.24 | 34 | 3.55 | 64 | 6.73 | 123 | 10.90 | 112 | 11.89 | 44 | 15.94 |
| severe | 0 | 0.00 | 5 | 0.69 | 12 | 1.25 | 15 | 1.58 | 37 | 3.28 | 25 | 2.65 | 13 | 4.71 |
| unable | 3 | 0.66 | 3 | 0.41 | 6 | 0.63 | 8 | 0.84 | 12 | 1.06 | 6 | 0.64 | 1 | 0.36 |
| **Pain/discomfort** |  |  |  |  |  |  |  |  |  |  |  |  |  |  |
| no | 330 | 72.05 | 491 | 67.82 | 590 | 61.52 | 464 | 48.79 | 475 | 42.11 | 323 | 34.29 | 77 | 27.90 |
| slight | 89 | 19.43 | 175 | 24.17 | 261 | 27.22 | 322 | 33.86 | 408 | 36.17 | 387 | 41.08 | 107 | 38.77 |
| moderate | 31 | 6.77 | 49 | 6.77 | 75 | 7.82 | 117 | 12.30 | 175 | 15.51 | 192 | 20.38 | 65 | 23.55 |
| severe | 4 | 0.87 | 9 | 1.24 | 24 | 2.50 | 34 | 3.58 | 52 | 4.61 | 33 | 3.50 | 23 | 8.33 |
| extreme | 4 | 0.87 | 0 | 0.00 | 9 | 0.94 | 14 | 1.47 | 18 | 1.60 | 7 | 0.74 | 4 | 1.45 |
| **Anxiety/depression** |  |  |  |  |  |  |  |  |  |  |  |  |  |  |
| no | 330 | 72.05 | 478 | 66.02 | 657 | 68.51 | 608 | 63.93 | 733 | 64.98 | 625 | 66.35 | 180 | 65.22 |
| slight | 81 | 17.69 | 173 | 23.90 | 204 | 21.27 | 207 | 21.77 | 254 | 22.52 | 225 | 23.89 | 66 | 23.91 |
| moderate | 31 | 6.77 | 55 | 7.60 | 61 | 6.36 | 90 | 9.46 | 95 | 8.42 | 76 | 8.07 | 21 | 7.61 |
| severe | 13 | 2.84 | 14 | 1.93 | 24 | 2.50 | 31 | 3.26 | 31 | 2.75 | 6 | 0.64 | 8 | 2.90 |
| extreme | 3 | 0.66 | 4 | 0.55 | 13 | 1.36 | 15 | 1.58 | 15 | 1.33 | 10 | 1.06 | 1 | 0.36 |
