# Supplementary material for: Population norms for the EQ-5D-5L for Hungary: comparison of online surveys and computer assisted personal interviews
Source: Eur J Health Econ. 2025 Feb 21;26(6):1111–26. doi: 10.1007/s10198-024-01755-2 (PMC12310892; doi:10.1007/s10198-024-01755-2)
Supplement: Supplementary file 7 — Supplementary Material 7 [file 10198_2024_1755_MOESM7_ESM.docx]

Online Resource 7 EQ-5D-5L index values by sex in the total sample and the two pooled samples

| Total | | | | | | |
| --- | --- | --- | --- | --- | --- | --- |
|  | Male | | | Female | | |
|  | N | Mean | (SD) | N | Mean | (SD) |
| **Total sample** | 4 886 | 0.89 | 0.2 | 5 438 | 0.88 | 0.2 |
| **Age group** |  |  |  |  |  |  |
| 18-24 | 248 | 0.95 | 0.13 | 458 | 0.94 | 0.14 |
| 25-34 | 463 | 0.95 | 0.14 | 724 | 0.94 | 0.11 |
| 35-44 | 881 | 0.93 | 0.17 | 959 | 0.91 | 0.18 |
| 45-54 | 899 | 0.9 | 0.22 | 951 | 0.87 | 0.22 |
| 55-64 | 976 | 0.86 | 0.22 | 1 128 | 0.84 | 0.24 |
| 65-74 | 1 098 | 0.87 | 0.18 | 942 | 0.84 | 0.19 |
| 75+ | 321 | 0.81 | 0.24 | 276 | 0.78 | 0.27 |
| **Education** |  |  |  |  |  |  |
| primary | 1 806 | 0.87 | 0.23 | 1 781 | 0.84 | 0.24 |
| secondary | 1 765 | 0.9 | 0.19 | 2 301 | 0.89 | 0.18 |
| tertiary | 1 315 | 0.91 | 0.15 | 1 356 | 0.9 | 0.17 |
| Pooled online surveys | | | | | | |
|  | Male | | | Female | | |
|  | N | Mean | (SD) | N | Mean | (SD) |
| **Total sample** | 3 406 | 0.87 | 0.21 | 3 898 | 0.86 | 0.21 |
| **Age group** |  |  |  |  |  |  |
| 18-24 | 104 | 0.91 | 0.18 | 297 | 0.92 | 0.16 |
| 25-34 | 238 | 0.91 | 0.19 | 478 | 0.92 | 0.12 |
| 35-44 | 576 | 0.91 | 0.19 | 685 | 0.88 | 0.21 |
| 45-54 | 660 | 0.88 | 0.25 | 694 | 0.84 | 0.24 |
| 55-64 | 713 | 0.84 | 0.24 | 882 | 0.82 | 0.25 |
| 65-74 | 896 | 0.87 | 0.18 | 720 | 0.84 | 0.20 |
| 75+ | 219 | 0.84 | 0.19 | 142 | 0.87 | 0.14 |
| **Education** |  |  |  |  |  |  |
| primary | 1 005 | 0.84 | 0.25 | 1 136 | 0.82 | 0.25 |
| secondary | 1 274 | 0.88 | 0.21 | 1 712 | 0.87 | 0.20 |
| tertiary | 1 127 | 0.9 | 0.16 | 1 050 | 0.89 | 0.18 |
| Pooled CAPI surveys | | | | | | |
|  | Male | | | Female | | |
|  | N | Mean | (SD) | N | Mean | (SD) |
| **Total sample** | 1 480 | 0.93 | 0.16 | 1 540 | 0.92 | 0.17 |
| **Age group** |  |  |  |  |  |  |
| 18-24 | 144 | 0.99 | 0.06 | 161 | 0.99 | 0.06 |
| 25-34 | 225 | 0.99 | 0.03 | 246 | 0.98 | 0.09 |
| 35-44 | 305 | 0.96 | 0.14 | 274 | 0.97 | 0.09 |
| 45-54 | 239 | 0.97 | 0.09 | 257 | 0.94 | 0.12 |
| 55-64 | 263 | 0.92 | 0.17 | 246 | 0.9 | 0.17 |
| 65-74 | 202 | 0.86 | 0.18 | 222 | 0.86 | 0.16 |
| 75+ | 102 | 0.73 | 0.3 | 134 | 0.67 | 0.33 |
| **Education** |  |  |  |  |  |  |
| primary | 801 | 0.91 | 0.19 | 645 | 0.87 | 0.22 |
| secondary | 491 | 0.96 | 0.11 | 589 | 0.94 | 0.14 |
| tertiary | 188 | 0.97 | 0.1 | 306 | 0.96 | 0.09 |
