# Supplementary material for: Population norms for the EQ-5D-5L for Hungary: comparison of online surveys and computer assisted personal interviews
Source: Eur J Health Econ. 2025 Feb 21;26(6):1111–26. doi: 10.1007/s10198-024-01755-2 (PMC12310892; doi:10.1007/s10198-024-01755-2)
Supplement: Supplementary file 8 — Supplementary Material 8 [file 10198_2024_1755_MOESM8_ESM.docx]

Online Resource 8 EQ VAS scores by sex in the total sample and the two pooled samples

| Total | | | | | | | |
| --- | --- | --- | --- | --- | --- | --- | --- |
|  | Male | | | Female | | | |
|  | N | Mean | (SD) | N | | Mean | (SD) |
| **Total sample** | 4 886 | 77.13 | 19.38 | 5 438 | | 76.89 | 19.71 |
| **Age group** |  |  |  |  | |  |  |
| 18-24 | 248 | 89.99 | 12.69 | 458 | | 84.24 | 17.11 |
| 25-34 | 463 | 85.65 | 16.31 | 724 | | 82.52 | 17.97 |
| 35-44 | 881 | 80.77 | 18.22 | 959 | | 79.20 | 19.73 |
| 45-54 | 899 | 78.92 | 18.93 | 951 | | 77.38 | 19.30 |
| 55-64 | 976 | 73.10 | 19.83 | 1 128 | | 73.98 | 20.36 |
| 65-74 | 1 098 | 72.59 | 19.05 | 942 | | 72.43 | 19.21 |
| 75+ | 321 | 67.67 | 19.35 | 276 | | 67.36 | 18.85 |
| **Education** |  |  |  |  | |  |  |
| primary | 1 806 | 75.38 | 21.26 | 1 781 | | 72.17 | 21.59 |
| secondary | 1 765 | 78.68 | 18.75 | 2 301 | | 78.67 | 18.83 |
| tertiary | 1 315 | 77.46 | 17.19 | 1 356 | | 80.08 | 17.33 |
| Pooled online surveys | | | | | | | |
|  | Male | | | Female | | | |
|  | N | Mean | (SD) | N | | Mean | (SD) |
| **Total sample** | 3 406 | 75.00 | 19.72 | 3 898 | | 75.41 | 20.08 |
| **Age group** |  |  |  |  | |  |  |
| 18-24 | 104 | 84.57 | 14.81 | 297 | | 79.73 | 17.54 |
| 25-34 | 238 | 80.23 | 19.15 | 478 | | 78.61 | 18.76 |
| 35-44 | 576 | 77.46 | 19.48 | 685 | | 76.39 | 21.18 |
| 45-54 | 660 | 76.52 | 19.83 | 694 | | 75.32 | 20.17 |
| 55-64 | 713 | 71.89 | 20.49 | 882 | | 73.63 | 20.97 |
| 65-74 | 896 | 73.26 | 19.09 | 720 | | 73.16 | 19.80 |
| 75+ | 219 | 70.97 | 18.91 | 142 | | 73.82 | 15.96 |
| **Education** |  |  |  |  | |  |  |
| primary | 1 005 | 72.94 | 22.31 | 1 136 | | 70.78 | 22.15 |
| secondary | 1 274 | 75.73 | 19.36 | 1 712 | | 76.82 | 19.38 |
| tertiary | 1 127 | 76.01 | 17.40 | 1 050 | | 78.14 | 17.90 |
| Pooled CAPI surveys | | | | | | | |
|  | Male | | | | Female | | |
|  | N | Mean | (SD) | | N | Mean | (SD) |
| **Total sample** | 1 480 | 82.03 | 17.62 | | 1 540 | 80.64 | 18.24 |
| **Age group** |  |  |  | |  |  |  |
| 18-24 | 144 | 93.91 | 9.14 | | 161 | 92.56 | 12.61 |
| 25-34 | 225 | 91.37 | 9.82 | | 246 | 90.11 | 13.42 |
| 35-44 | 305 | 87.03 | 13.54 | | 274 | 86.24 | 13.16 |
| 45-54 | 239 | 85.56 | 14.22 | | 257 | 82.96 | 15.46 |
| 55-64 | 263 | 76.38 | 17.54 | | 246 | 75.23 | 18.00 |
| 65-74 | 202 | 69.60 | 18.64 | | 222 | 70.04 | 16.97 |
| 75+ | 102 | 60.58 | 18.44 | | 134 | 60.51 | 19.32 |
| **Education** |  |  |  | |  |  |  |
| primary | 801 | 78.44 | 19.45 | | 645 | 74.62 | 20.36 |
| secondary | 491 | 86.32 | 14.52 | | 589 | 84.05 | 15.97 |
| tertiary | 188 | 86.10 | 12.83 | | 306 | 86.75 | 13.23 |
